# Annotations

**Published:** 1907-08-24

**Authors:** 


### ANNOTATIONS.

### The Chloroform Controversy.

The recent discussion on "Anæsthetics" at the British Association has elicited from Lieut.-Col. Lawrie an interesting statement on the historical aspect of the chloroform controversy. In a letter to the *Times*, Col. Lawrie defines the position of the "old Edinburgh school," and quotes the directions which Syme used to give for successful chloroform administration. He states that Syme never had a death from chloroform, and that those who follow his principles have given chloroform "all over the world with the same safety and success that he had." Syme's directions may be summarised as follows. A free admixture of air with the vapour of chloroform, which is ensured by administration from some such material as a folded towel or handkerchief; if this is attended to, the more rapidly the anæsthetic is given the better until the effect is produced; guidance is obtained not from the pulse, but by watching the respiration; no apparatus is used, and administration should never be continued beyond the point where the patient is fully under the influence of the anæsthetic. It was in 1889, and at Col. Lawrie's suggestion, that the Nizam of Haidarabad appointed the celebrated Haidarabad Commission, and this, it is contended, "proved conclusively and finally that chloroform, when given by inhalation, does not affect the heart directly," and established the truth of Syme's principles "upon an absolutely definite scientific basis." This, Col. Lawrie maintains, is a plain statement of an established fact, which can be demonstrated in any laboratory or operating-theatre as a proof that, when chloroform is given on the above principles, "danger and death are alike impossible." If physiologists continue to regard chloroform as a dangerous drug, this merely shows that the work of the Haidarabad Commission has been neglected. The interesting question, however, is, that, assuming all these contentions to be accurate, why have Syme's teaching and method not commanded universal adoption?

### The Medicinal Treatment of Cancer.

Not the least interesting part of the Cancer Research Report is that which deals with the subject of treatment. The findings of the investigators are interesting, but at the same time they are terribly disappointing, because the record which they furnish is entirely negative. The claim that trypsin, either alone or in conjunction with amylopsin, had cured two mice inoculated with portions of Jensen's tumour was tested by a series of experiments, and after a very careful study of results the verdict is that the alleged remedy was found to be useless. This judgment on a method of treatment which, with all its limitations, appeared at least to offer a more hopeful chance of success than any other method of medicinal treatment is, of course, disheartening, but disappointing though the facts are, they will have to be faced. The treatment of cancer by pancreatic ferments has had a careful and unprejudiced trial, and has been found useless. It must, however, be asked whether the facts elicited by this inquiry mean a complete abandonment of the use of medicinal agents in the treatment of the disease. As a substitute for early and effective operation, such agents, it must be admitted, have at present no field of usefulness. But in inoperable cases there is surely reason to continue careful observation and experiment. The situation in such cases cannot be worse, and in a certain number of instances there is reason to believe that it may be alleviated. In some quarters unwarranted "booms" have obviously been cultivated, but there are certain careful and conscientious men who have already gained some encouragement, and we sincerely trust that they will continue their work.

### Medical Sickness and Accident Assurance.

It is a frequent taunt that members of the medical profession are not men of affairs, and that they often fail to arrange both their corporate aims and their personal interests on a sound business basis. In all this it must be admitted there is some measure of truth, and when the circumstances of professional training are considered the absence of the sound commercial instinct is not altogether a matter for wonder. Failure in this respect is occasionally illustrated in pathetic fashion when a practitioner's health breaks down and it is found that he has made no provision to meet any such misfortune. To him and to those dependent on him the consequences of such a position are apt to be disastrous. Medical practice is so largely, often indeed entirely, a personal matter, that a practitioner's ill-health or sickness means an absolute cessation of income. Hence there is no class on whose members it is more incumbent to avail themselves of every opportunity to secure provision against failure of health than the medical profession. To do this by personal effort and economy is often impossible, more especially in the earlier years of life, and hence it is wise to consider what may be effected by the principles of co-operation and mutual insurance. In this connection we venture to direct the attention of our readers to the Medical Sickness and Accident Assurance Society, the offices of which are at 33 Chancery Lane, W.C. The Society has been in existence since 1884, and the membership, which is entirely confined to the medical and dental professions, has grown from 200 to 2,488. A sum of more than £100,000 has been paid in response to claims from policy-holders. A policy will secure a payment of four guineas a week during professional disablement, and in cases of permanent incapacity half this sum will be continued to the age of 65 years. No payments are made for commissions and no agents are employed, and in the course of some 20 years over £12,000 has been returned in the shape of cash bonuses to members. The chairman of the Society is Dr. de Havilland Hall, and the trustees include Sir Dyce Duckworth, Sir Victor Horsley, Dr. Heron, and Dr. Dawson Williams. Here, as in so many other directions, united action by the profession would secure both a more powerful institution and increased personal benefits to individuals. We are glad to have the opportunity of commending this valuable professional organisation to the attention of our readers.

